# Successful management of pyriform sinus cyst and fistula using endoscopic electrocauterization

**DOI:** 10.1002/deo2.128

**Published:** 2022-05-15

**Authors:** Naonori Kawakubo, Satoshi Obata, Koichiro Yoshimaru, Kina Miyoshi, Tomoko Izaki, Tatsuro Tajiri

**Affiliations:** ^1^ Department of Pediatric Surgery Faculty of Medical Sciences Kyushu University Fukuoka Japan; ^2^ Department of Pediatric Surgery Miyazaki Prefectural Hospital Miyazaki Japan; ^3^ Department of Pediatric Surgery Oita Prefectural Hospital Oita Japan

**Keywords:** electrocauterization, endoscopic cauterization, pediatric, pyriform sinus cyst, pyriform sinus fistula

## Abstract

**Objectives:**

Pyriform sinus cyst (PSC) and pyriform sinus fistula (PSF) is a rare congenital malformation that arises from the third or fourth branchial structure. In our study, we describe the safety and the utility of endoscopic electrocauterization against PSC/PSF.

**Methods:**

We retrospectively reviewed the records of patients who underwent endoscopic electrocauterization for PSC/PSF at our hospital. The internal opening of the fistula was identified under general anesthesia using a flexible endoscope (XQ‐260 or H‐290; Olympus, Tokyo, Japan), and the DualKnifeJ (KD‐655L; Olympus) was used to ablate the internal opening.

**Results:**

We experienced three PSF and three PSC patients. The postoperative course was uneventful in all cases. The patients declared no pain in the neck, and there were no cases showing recurrent nerve paralysis. Five in six cases (83%), the closure of fistula was archived in the first cauterization. One case (16.6%) required repeated cauterization. No recurrence was found during the follow‐up period ( median: 1 year) in any cases.

**Conclusions:**

Owing to its rarity in neonates, the diagnosis and treatment of PSC remains complicated and not clearly described. Complete removal of the fistula and the cyst with or without affected thyroid tissue was previously the most commonly used treatment. From our experience, we believe that endoscopic electrocauterization can be the first choice not only for PSF but also for neonatal PSC. In conclusion, endoscopic electrocauterization is feasible even for neonatal PSC. Further investigations including multicenter analyses are needed.

## INTRODUCTION

Pyriform sinus cyst (PSC) and pyriform sinus fistula (PSF) are rare congenital malformations that arise from the third or fourth branchial structure.^1^ Complete removal of the fistula and cyst with or without affected thyroid tissue was previously the most commonly used and most thorough treatment.^2^ However, severe scarring due to recurrent abscesses or repetitive incisions and drainage can increase the risk of developing postoperative complications.^1^

An alternative procedure for finding an opening in PSC/PSF and performing endoscopic chemical cauterization with trichloroacetic acid (TCA) as the first‐line treatment for PSF with a combination of endoscopy and treatment using rigid laryngoscopy was recently reported.^3^, ^4^ This approach can reduce the incidence of postoperative complications and has a higher cure rate than open surgery. However, transient paralysis of the vocal cords after TCA chemical cauterization has been reported.^5^ The most popular endoscopic electrocautery technique was first described in 1998,^6^ and laser ablation has also been reported as a minimally invasive treatment for PSF.^7^ The basic principles of chemical ablation and electrocauterization are aimed at circumferential ablation of internal openings. Lachance et al. reported the systematic review of the endoscopic obliteration technique for managing PSF, and the endoscopic management appeared to be safe and effective.^8^ In Japan, the reports about the endoscopic obliteration technique for PSF are few. Moreover, in past, there was only one report that focused on endoscopic treatment for neonatal PSC.^9^ In the present study, we describe the safety and utility of endoscopic electrocauterization for pediatric PSC/PSF. We actively treated neonatal PSC using endoscopic electrocauterization with a focus on treatment for neonatal PSC.

## PATIENTS AND METHODS

We retrospectively reviewed the records of patients who underwent endoscopic electrocauterization of PSC/PSF at Kyushu University Hospital from January 2017 to January 2022.

Since 2017, we have actively treated PSC/PSF by endoscopic electrocauterization. The internal opening of the fistula was identified under general anesthesia using a flexible endoscope (GIF‐XQ260 or GIF‐H290; Olympus, Tokyo, Japan), and the DualKnifeJ (KD‐655L; Olympus) was used to ablate the internal opening of the pyriform sinus; the modes of electrocauterization were FORCED COAG mode (Effect 2, 10–20 W), and the electrocautery unit was VIO 300D (ERBE). When the patients underwent Sclerotherapy using OK‐432, the surgeon performed an ultrasound‐guided puncture to the cyst and injected 5 ml of OK‐432 (0.1 KE/ml). And, almost 1 h after the sclerotherapy, the injected OK‐432 was collected from the cyst through the inserted needle. During this procedure, the patient was in the supine position, and no laryngeal elevation was required.

The institutional review board of our hospital approved this study. This retrospective study was performed according to the Ethical Guidelines for Clinical Research, published by the Ministry of Health, Labour and Welfare, Japan, on July 30, 2003 (revised in 2008). The study complied with the 1964 Declaration of Helsinki (revised in 2008). The Institutional Review Board exempted this study from the need for informed consent.

## RESULTS

During the above period, we experienced three PSF and three PSC patients. In all of the patients, the PSF and PSC were located on the left side. There was no other patient who underwent other treatment (e.g., surgery) for PSF/PSC during the same period.

**FIGURE 1:**
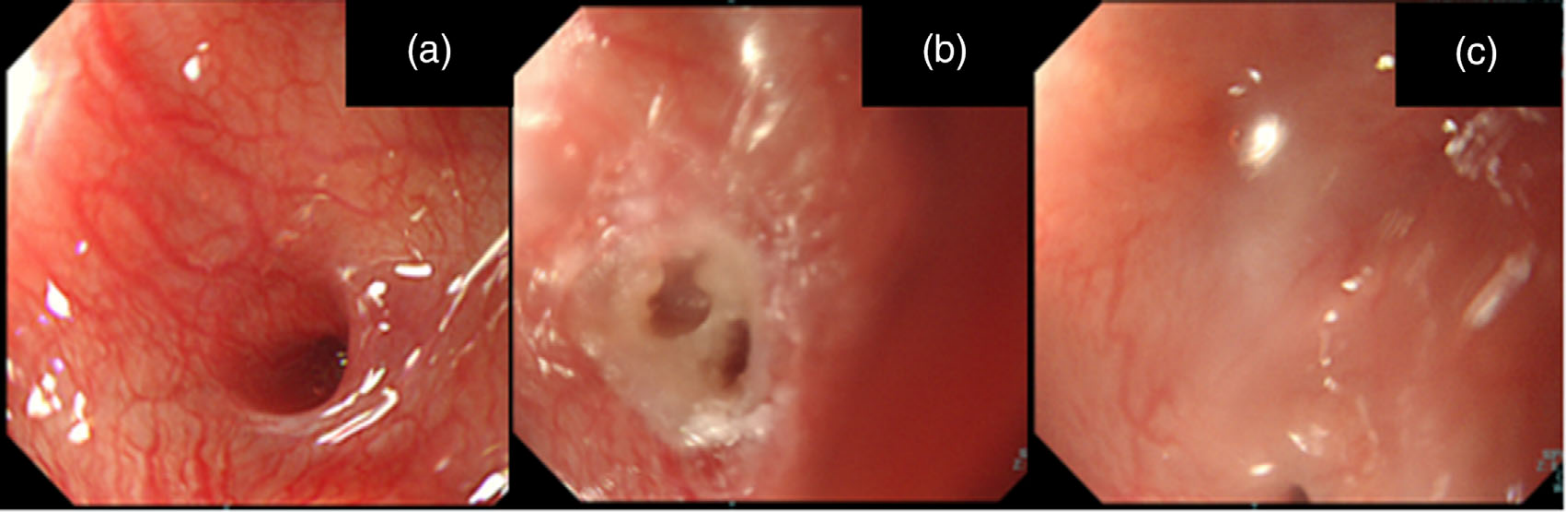
Endoscopic findings of pyriform sinus cyst. Case 6 (a–c). (a) The internal opening of the pyriform sinus (Case 6). (b) The internal opening after ablation. (c) The finding of endoscopy 2 weeks after ablation. Closure of the fistula was observed

The details of the six patients are summarized in Table 1. There were no intraoperative complications. The endoscopic findings are listed in Figure 1 (Case 6). For two of the three PSC patients, sclerotherapy using OK‐432 was performed after endoscopic electrocauterization, and the other patient underwent only endoscopic electrocauterization. Regarding the three PSF patients, one (Case 5) who had an acute inflammatory neck tumor underwent an external incision and drainage of the neck abscess.

**TABLE 1:** Results of six patients

**Case**	**Age**	**Sex**	**Number of procedures**	**Operative time (min)**	**Combination therapy**	**Left or Right**	**Cyst**	**Follow‐up period**
1	1 month	Male	1	94	Sclerotherapy	Left	Yes	4 years
2	18 days	Female	3	34/44/63	Sclerotherapy	Left	Yes	4 years
3	12 years	Female	1	15	‐	Left	No	3 years
4	2 years	Male	1	11	‐	Left	No	1 year
5	4 years	Male	1	29	Drainage of abscess	Left	No	1 year
6	1 month	Male	1	10	‐	Left	Yes	3 months

**FIGURE 2:**
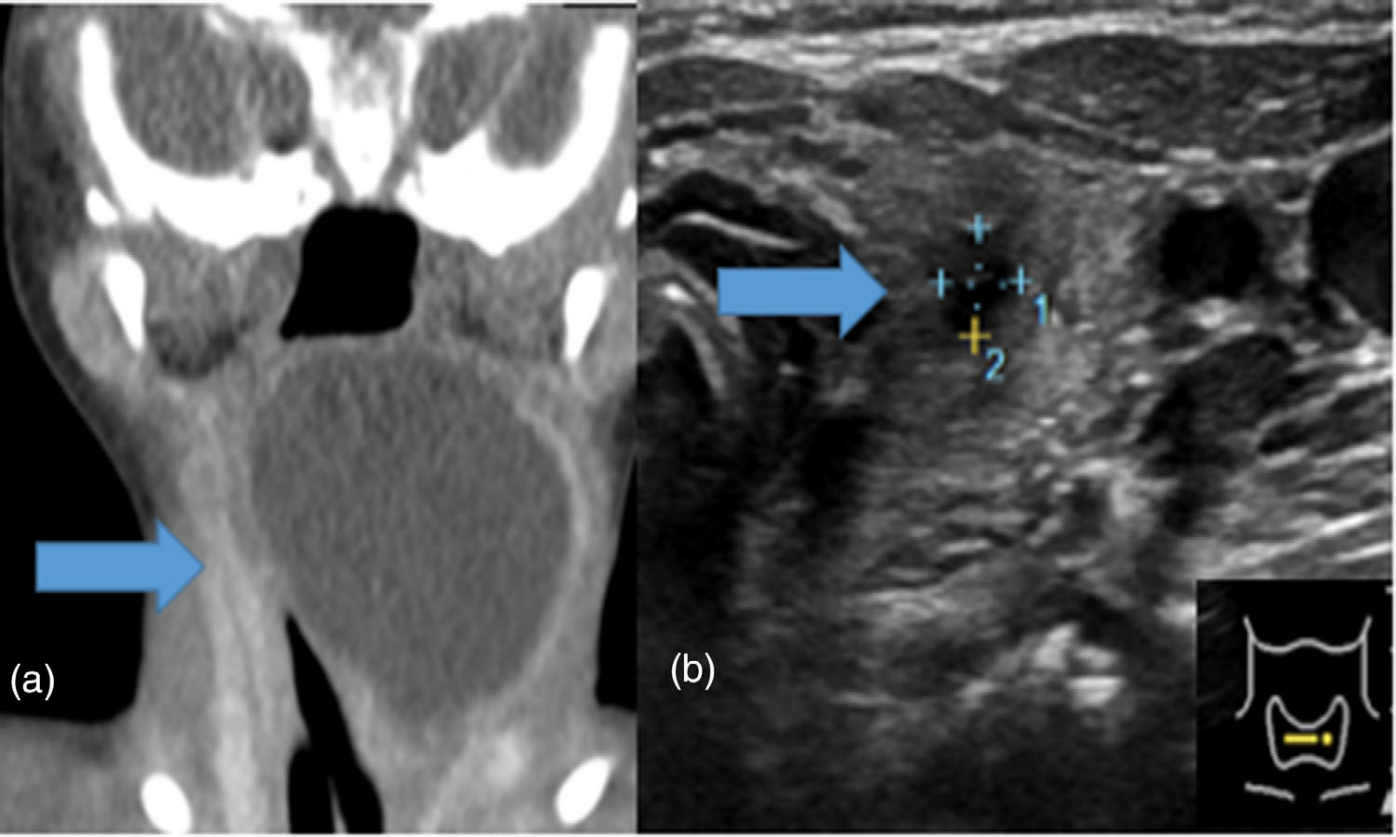
Computed tomography and ultrasonography findings of pyriform sinus cyst before and after ablation. Case 6 (a, b). (a) Computed tomography findings before ablation. A large cystic mass occupies the left side of the neck. (b) Ultrasonography findings one month after ablation. The cystic mass has remarkably shrunk

The postoperative course was uneventful in all cases. The patients declared no pain in the neck, and there were no cases of recurrent nerve paralysis. Five in six cases (83%), the closure of fistula was archived in the first cauterization. Case 2 required repeated cauterization (three times), but the fistula was ultimately closed after repeated cauterization. The cyst of the neck shrank but still remained in two patients (Cases 1 and 2), and we have continued follow‐up of these patients. In one patient (Case 6), the cyst shrank remarkably, and we were able to detect a tiny cyst (3 mm) on ultrasonography (Figure 2). No cases of recurrence were found during the follow‐up period (median: 1 year).

## DISCUSSION

PSF and PSC are rare congenital branchial arch anomalies in children.^1^ PSC is commonly identified during the newborn period and causes respiratory failure, neck abscess, or thyroiditis.^1^, ^2^, ^10^ It originates from the failure obliteration of the third or fourth pharyngeal pouches, which are connected to the pharynx by the pharyngobranchial duct.^1^, ^2^ Owing to its rarity in neonates, the diagnosis and treatment of PSC remains complicated and not clearly described. Complete removal of the fistula and cyst with or without affected thyroid tissue was previously the most commonly used and most thorough treatment.^1^, ^2^, ^10^

Recently, however, studies involving ablation of the internal opening for treatment of PSF have been reported.^11^, ^12^ Various ablation techniques have been described, including TCA^3^, ^4^ ^,^ electrocauterization,^6^, ^13^, ^14^ radiofrequency ablation,^15^ laser cauterization,^7^ ^,^ and others.^16^ Chen et al. reported the utility of endoscopic radiofrequency ablation compared to endoscopic‐assisted surgery.^15^ In their report, the patients treated with radiofrequency ablation had a significantly shorter hospital stay than those treated with endoscopic‐assisted surgery. Hwang et al. reported a retrospective analysis comparing excision versus TCA.^3^ In their report, TCA chemocauterization was performed for 13 patients, and the recurrence rate was 46.1%. All of the patients with recurrence were successfully treated with repeated chemocauterization or re‐excision. A large cohort of endoscopic electrocauterization was initially reported by Chen et al.^13^. They used the Bugbee flexible cautery electrode and inserted the electrode into the sinus tract and ablate the tract. They advocated this procedure as a first‐line treatment in conjunction with incision and drainage. In another report that emphasized the utility of electrocauterization,^14^ authors considered that cauterization was a proper first‐line treatment for PSF.

Electrocauterization of PSC for neonatal patients is a more challenging procedure than electrocauterization for older patients. Two of our three patients underwent sclerotherapy using OK‐432 to ablate the internal epithelium of the cyst, but the cysts remained in both cases. We considered sclerotherapy for PSC not to be very effective or necessary. Thus, in Case 6, the patient underwent only electrocauterization. The internal openings were closed in all cases, and the cysts remained in two cases, but in one case (treated with only electrocauterization), the cyst shrank remarkably. In our experience, closure of the internal opening of the fistula is the most important factor for managing PSC as well as PSF.

We should consider the risk for each obliteration technique. In chemical ablation such as TCA, there is a risk of oral inflammation for the spillage of chemical agents to another oral cavity. In radiofrequency ablation or laser ablation, postoperative temporary hoarseness occurred in 11.1% of patients.^15^ The postoperative hoarseness is often observed even after the open surgery to PSF/PSC, and we should pay attention to the overcauterization of the internal opening. As Ishinaga et al. reported,^14^ the Bugbee cautery electrode can be inserted 1–2 cm deep into a fistula, but we feel that blind cauterization is dangerous, considering the risk of damaging the recurrent laryngeal nerve. We ablated the internal opening using Dualknife J. Because the internal opening of the pediatric (especially neonatal) pyriform sinus is very small, a fine operation is needed, and we think Dualknife J is a suitable device to ablate in such a small area. We think the advantage of this procedure is targeted electrocauterization under direct endoscopic observation, which allows for effective cauterization minimizing the cautery effect on surrounding organs.

In Table 2, we summarized the reports about the obliteration technique for managing PSF/PSC. As mentioned above, when TCA was used, a postoperative complication such as temporary vocal fold immobility occasionally occurred. There was only one report about endoscopic electrocauterization for neonatal PSC.^8^ In that report, the high rate of failure of endoscopic treatment (40% 2/5) was reported. Further investigation is needed if endoscopic treatment is truly effective for neonatal PSC, but we think the electrocauterization of the internal opening of the fistula is effective not only for PSF but neonatal PSC.

**TABLE 2:** The summary of obliteration technique for pyriform sinus fistula/pyriform sinus cyst

**Author**	**Patient number**	**Obliteration technique**	**Success rate(%)**	**Complication rate(%)**	**Age. (range mo)**
Kim^17^	11	TCA	81.8	0	24–144
Stenquist^18^	1	TCA	100	0	166
Cigliano^19^	1	Fibrin glue	100	0	108
Sayadi^20^	2	CERALAS laser	100	0	36–144
Ahmed^21^	3	Electrocautery	100	0	12–84
Pereira^22^	2	Silver nitrate	100	0	24–204
Chen^13^	9	Electrocautery	78	0	49–193
Leboulanger^9^	19	CO_2_ laser, thulium laser, electrocautery	84.2	0	0–204
Park^5^	2	TCA	100	100	13–60
Watson^23^	5	Electrocautery, CO_2_ laser plus silver nitrate, silver nitrate	100	0	36–144
Sun^24^	22	Electrocautery	95.5	0	6–124
Cha^25^	44	TCA	77.3	0	24–648
Wong^26^	2	Electrocautery	100	0	132–168
Hwang^3^	13	TCA	54	0	18–180
Yanagisawa^4^	4	TCA	100	50	60–96
Wang^7^	101	CO_2_ laser,	97	0	13–197
Masuoka^12^	74	TCA, diode laser	83 (TCA) 100 (laser)	5.4	60–552
Ishinaga^14^	8	Electrocautery	75%	0	36–636
Chen^15^	48	Radioablation	100	13.2	33–97
Xia^16^	10	Hypothermia plasma	100	0	NA
Hod^27^	10	Electrocautery plus voicegel	100	0	12–84
Present cases	6	Electrocautery, electrocautery plus sclerotherapy	100	0	0–170

TCA, trichloroacetic acid

In the present study, we cauterized only the mucous membrane of the internal opening endoscopically. Careful consideration should be given to the effects of residual cysts. Based on our experience, we believe that this procedure can be the first choice for not only PSF but also newborn PSC.

In conclusion, endoscopic electrocauterization is a viable procedure, even for neonatal PSC. However, our study was associated with some limitations, including its retrospective design, and further investigations that include multicenter analyses are needed.

## CONFLICT OF INTEREST

The authors declare no conflict of interest.

## FUNDING INFORMATION

None.
